# Phage reprogramming of *Pseudomonas aeruginosa* amino acid metabolism drives efficient phage replication

**DOI:** 10.1128/mbio.02466-24

**Published:** 2025-02-07

**Authors:** Alexa D. Fitzpatrick, Véronique L. Taylor, Pramalkumar H. Patel, Dominick R. Faith, Patrick R. Secor, Karen L. Maxwell

**Affiliations:** 1 Department of Biochemistry, University of Toronto, Toronto, Canada; 2 Department of Microbiology and Cell Biology, Montana State University, Bozeman, Montana, USA; Freie Universitat Berlin, Berlin, Germany

**Keywords:** phage infection, bacterial metabolism, biofilm, *Pseudomonas aeruginosa*

## Abstract

Phages have been shown to use diverse strategies to commandeer bacterial host cell metabolism during infection. However, for many of the physiological changes in bacteria during infection, it is often unclear if they are part of a bacterial response to infection or if they are actively driven by the phage itself. Here, we identify two phage proteins that promote efficient phage replication by reprogramming host amino acid metabolism. These proteins, Eht1 and Eht2, are expressed early in the infection cycle and increase the levels of key amino acids and the arginine-derived polyamine putrescine. This provides a fitness advantage as these metabolites are important for phage replication and are often depleted during infection. We provide evidence that Eht1 and Eht2 alter the expression of bacterial host metabolic genes, and their activities may impinge on metabolism-related signaling processes. This work provides new insight into how phages ensure access to essential host resources during infection and the competitive advantage this provides.

**IMPORTANCE:**

Bacterial viruses, known as phages, are abundant in all environments that are inhabited by bacteria. During the infection process, phages exploit bacterial resources, resulting in notable changes to bacterial metabolism. However, precise mechanisms underlying these changes, and if they are driven by the phage or are a generalized bacterial response to infection, remain poorly understood. We characterized two proteins in *Pseudomonas aeruginosa* phage JBD44 whose activities alter bacterial host metabolism to optimize phage replication. Our work provides insight into how phages control bacterial processes to ensure access to essential host resources during infection.

## INTRODUCTION

When phages infect bacteria, they must quickly recruit host nutrients and resources and overcome anti-phage defenses. Phages express a suite of proteins to aid in this process. Phage takeover of the bacterial cell is complex and multifaceted because bacteria harbor diverse anti-phage defenses that limit phage infection and have extensive gene regulatory networks that allow them to quickly respond to environmental changes (1, 2). Studies have shown that global changes to metabolism are common during phage infection and that phages can strategically reprogram host metabolism for their own purposes (3, 4). These changes can be mediated by phage-encoded genes, sometimes referred to as “auxiliary metabolic genes” (5), because they are thought to support key steps in bacterial metabolism that are needed for phage replication.

Different phages affect bacterial metabolic pathways in unique ways. For example, cyanophages infecting *Prochlorococcus* and *Synechococcus* encode proteins that direct carbon flux from the Calvin cycle to the pentose phosphate pathway to fuel deoxynucleotide biosynthesis for phage replication (6). Phages have also been shown to encode proteins that can sequester phosphate, thereby supporting replication in phosphate-starved cells (7). Some pathways, like those involved in amino acid metabolism, are altered during infection by many diverse phages. For example, amino acid pools are depleted, and expression patterns of genes involved in amino acid biosynthetic pathways are altered in *P. aeruginosa* cells during infection by phages PAK_P3, PaP1, YuA, 14-1, PEV2, LUZ19, and LUZ24 (3, 8). The metabolic pathway that controls arginine and proline production is closely associated to polyamine metabolism, and polyamines have been shown to bind nucleic acids and assist with phage replication, translation, and DNA packaging into capsids (9, 10). Arginine and polyamines also affect levels of cyclic-di-GMP, one of the most widespread and important cyclic nucleotide messengers across bacteria (11, 12). In turn, changes in c-di-GMP signaling impact phage replication through alterations to bacterial metabolism (13) and expression of phage receptors on the cell surface (14). There has been increasing evidence on how phages can tap into host amino acid metabolism and c-di-GMP signaling (15), but these are still underexplored aspects of bacteria–phage relationships. Notably, many studies do not clearly distinguish if changes to bacterial physiology are driven by phage proteins or are a result of a bacterial response to infection and whether these changes benefit the phage.

In this study, we characterized two proteins in *P. aeruginosa* phage JBD44 that are important for efficient and competitive phage replication. These proteins, which we call Eht1 and Eht2 for “early host takeover,” are expressed early in the infection cycle. Their expression alters amino acid metabolism and c-di-GMP levels, leading to increased levels of arginine and the arginine-derived polyamine putrescine. We found these metabolites can rescue a loss of infection efficiency in the absence of Eht1 and Eht2. JBD44 infection leads to differential expression of genes related to two-component systems that are key, membrane-localized regulators of metabolism and signaling in bacteria (16). As Eht1 and Eht2 are predicted to localize to the inner membrane, we propose that they drive these metabolic changes through interactions with two-component systems. This work provides new insight into the host takeover strategies used by phages during infection.

## RESULTS

**Fig 1:**
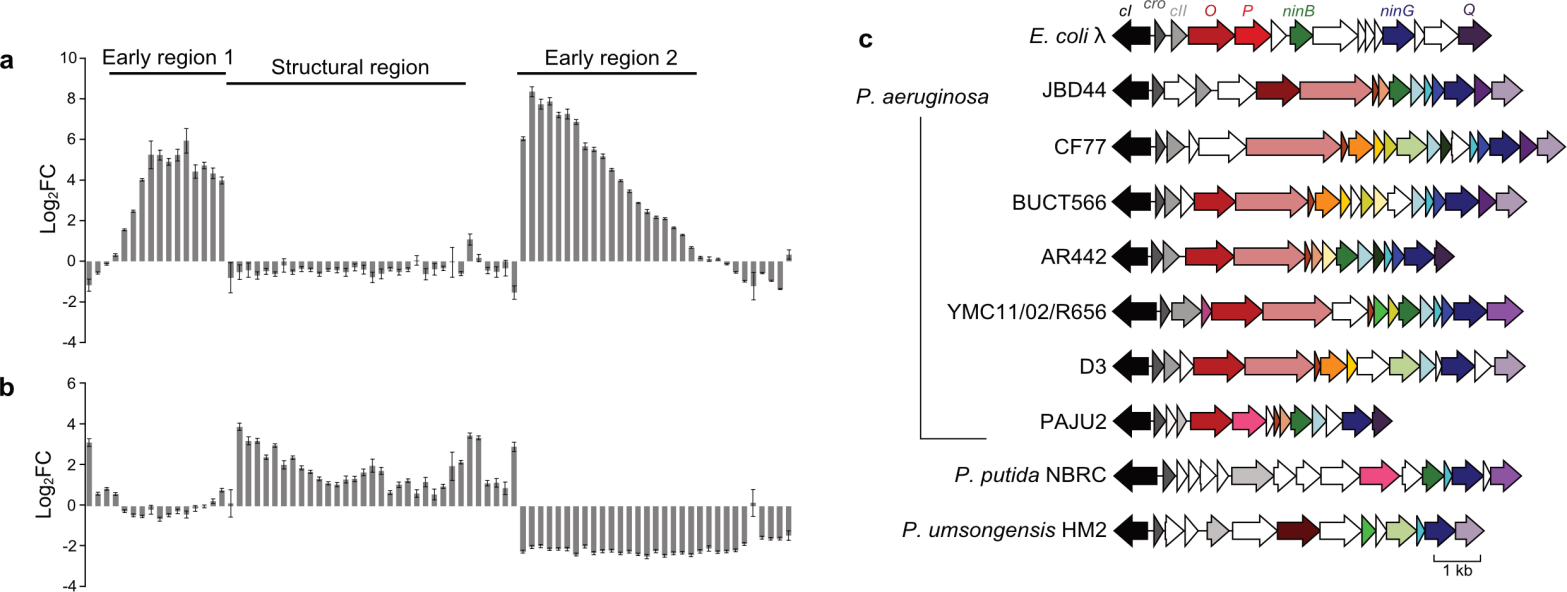
*Pseudomonas* phage JBD44 gene expression patterns during infection. Differential expression of JBD44 genes, each represented by an individual bar, across the genome with changes shown from (a) 2 to 5 minutes and (b) 5 to 20 minutes post-infection. *eht1* is highlighted in light blue and *eht2* in dark blue. Error bars represent the standard error of two biological replicates (*P* < 0.05, Wald test). (c) Genome alignment diagram of the early right operon comparing phage λ with various *Pseudomonas* phages. Each arrow represents an open reading frame, and genes that share >30% sequence identity are colored the same.

To identify genes that are expressed during early host takeover by phage JBD44, we performed an RNAseq experiment. We infected *P. aeruginosa* strain PA14 in the early exponential growth phase with JBD44 at a multiplicity of infection (MOI) of 10 to ensure synchronous infection of all cells. We took samples immediately before infection, and at 2, 5, and 20 minutes post-infection. We performed Illumina RNAseq and determined the average fold-change of gene expression from two independent replicates for both phage (Table S1), and bacterial (Table S2) genes. We identified two regions of the phage JBD44 genome that increased expression >fourfold from 2 to 5 minutes post-infection, which we called early regions 1 and 2 (Fig. 1a; Table S1). At 20 minutes post-infection, the genes in early region 1 were slightly downregulated as compared to 5 minutes post-infection, and the genes in region 2 were downregulated by >twofold (Fig. 1b; Table S1). Early region 1 encompasses genes 3 to 12. The genes found in this region are similar to those in the *Escherichia coli* phage λ early right operon (*p*_R_), which consists of a series of 12 overlapping genes that encode proteins known to be involved in early steps of phage infection, including replication initiation proteins O and P, and recombination proteins NinB and NinG (17, 18). This region in phage JBD44 consists of similarly tightly linked genes and encodes homologs of replication initiation proteins O, NinB, and NinG. We found this region is conserved in other *P. aeruginosa* phages including D3 (19), and PAJU2 (20) (Fig. 1c). Bioinformatic analyses revealed that different phages share different combinations of genes in this region (Fig. 1c), suggesting that these encode accessory proteins that may aid in the early steps of phage infection. The second upregulated region, which includes genes 46 to 64, resembles the phage λ early left operon (*p*_L_) and contains the integrase and an *ea22*-like gene. The region encoding genes 18 to 39, which is known to encode the proteins required for phage virion assembly, was upregulated >threefold from 5 to 20 minutes post-infection (Fig. 1b; Table S1). This timing of gene expression in JBD44 is consistent with those in both phage λ (21) and previously studied *P. aeruginosa* phages PaP1 (22) and PaP3 (8).

**Fig 2:**
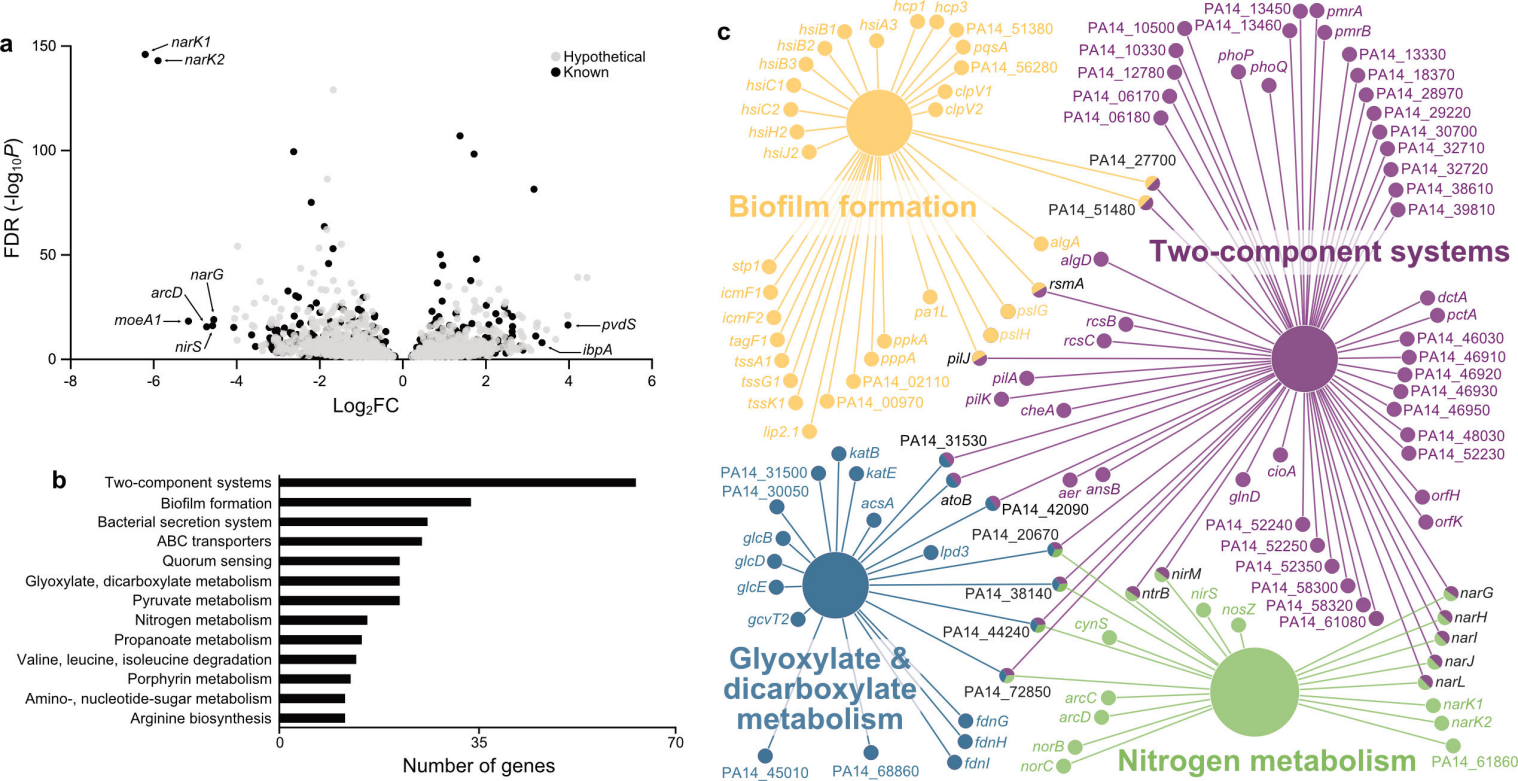
PA14 differential gene expression and pathway enrichment analysis for JBD44 infection. (a) Transcriptomic analysis of PA14 genes from 5 to 20 minutes post-infection by JBD44 (*P* < 0.05, Wald test). The log_2_FC indicates the mean expression level for each gene. Each dot represents one gene, with genes of unknown function shown in gray and annotated genes shown in black. (b) Analysis of the most common KEGG pathways associated with the differentially expressed PA14 genes, with the number of known genes in these pathways shown. (c) Network plot of the most enriched KEGG pathways associated with the differentially expressed PA14 genes using ClueGO enrichment analysis (Kappa score 0.4). Different pathways are shown in different colors, and the size of the node for each pathway is scaled to represent the enrichment of that pathway (*P* values in Table 1).

We next examined how JBD44 infection impacts the bacterial host by assessing changes in PA14 gene expression from 2 to 5 minutes and 5 to 20 minutes post-infection. We found ~2,500 genes were significantly differentially expressed (*P* < 0.05), with approximately equal numbers of genes upregulated and downregulated at both time points (Fig. 2a; Table S2). Genes involved in the two predominant anaerobic metabolic pathways, denitrification and arginine fermentation, were the most severely repressed. This included denitrification genes from the *narK1K2GHJHI* operon (23) and the *arcDABC* operon involved in arginine fermentation (24), which were both downregulated by >16-fold at both time points (Fig. 2a; Table S2). Genes involved in denitrification and arginine fermentation have previously been shown to be differentially expressed during phage infection (25), but how this impacted phage replication was not explored. We noted that genes involved in stress responses were upregulated. For example, the heat shock protein *ibpA* and the iron-starvation factor *pvdS* were both upregulated >8-fold by 5 minutes post-infection (Fig. 2a; Table S2). Other upregulated genes known to be connected with the stress response include the efflux pump operon *mexGHI-opmD* (26) and the chaperone *grpE* (Table S2). Genes involved in stress responses are commonly upregulated upon phage infection (8, 27).

To gain insight into how changes in gene expression during phage infection impact PA14 physiology, we grouped differentially expressed genes into their associated KEGG pathways using Cytoscape ClueGO (28). This analysis includes genes of known function as well as hypothetical proteins that are known to be associated with a pathway but have an unknown role. This is important because approximately 70% of the differentially expressed genes in *P. aeruginosa* encoded proteins of unknown function. To narrow in on the KEGG pathways most impacted by JBD44 infection, we looked for those that were enriched for genes upregulated or downregulated by >2-fold. We focused on PA14 genes that are differentially regulated from 5 to 20 minutes post-infection to allow time for the expression of early phage genes that have been shown to best correlate to changes in bacterial gene expression (8). We found many of the differentially expressed genes are associated with metabolic pathways (Fig. 2b). The two-component system pathway was the most common individual pathway, associated with >60 differentially expressed genes at both 5 and 20 minutes post-infection (Fig. 2b; Table S3). This is consistent with a known role for membrane-associated two-component systems in sensing external cues and mediating transcriptional regulation of pathways involved in response to these cues (16). We noted that many other differentially expressed genes at both time points were associated with pathways involving other membrane-localized functions including ABC transporters and secretion systems (Fig. 2b), which has previously been shown during phage infection (29).

Using KEGG pathway enrichment analyses in Cytoscape ClueGO (28), we identified four pathways that were significantly enriched for PA14 genes differentially expressed from 5 to 20 minutes post-infection by JBD44 (Kappa score 0.4): nitrogen metabolism, two-component systems, glyoxylate and dicarboxylate metabolism, and biofilm formation (Fig. 2c; Table 1). The nitrogen metabolism pathway was the most highly enriched, with ~50% of the genes found in this KEGG family pathway (map00910) being differentially expressed (Table 1). This includes highly repressed genes involved in denitrification and arginine fermentation (Table S3). Within the nitrogen metabolism pathway, ~75% of the genes are repressed (Table S3), suggesting that JBD44 largely represses nitrogen metabolism during infection.

**TABLE 1:** KEGG pathways enriched for differentially expressed PA14 genes from 5 to 20 minutes post-infection by JBD44

Term	KEGG pathway	Number of genes	Percent of pathway	Term *P*-value
Nitrogen metabolism	KEGG:00910	21	47.06	0.004
Glyoxylate and dicarboxylate metabolism	KEGG:00630	22	36.07	0.025
Biofilm formation	KEGG:02025	35	31.25	0.016
Two-component systems	KEGG:02020	65	26.64	0.009

### JBD44 genes *eht1* and *eht2* facilitate productive and competitive phage replication

**Fig 3:**
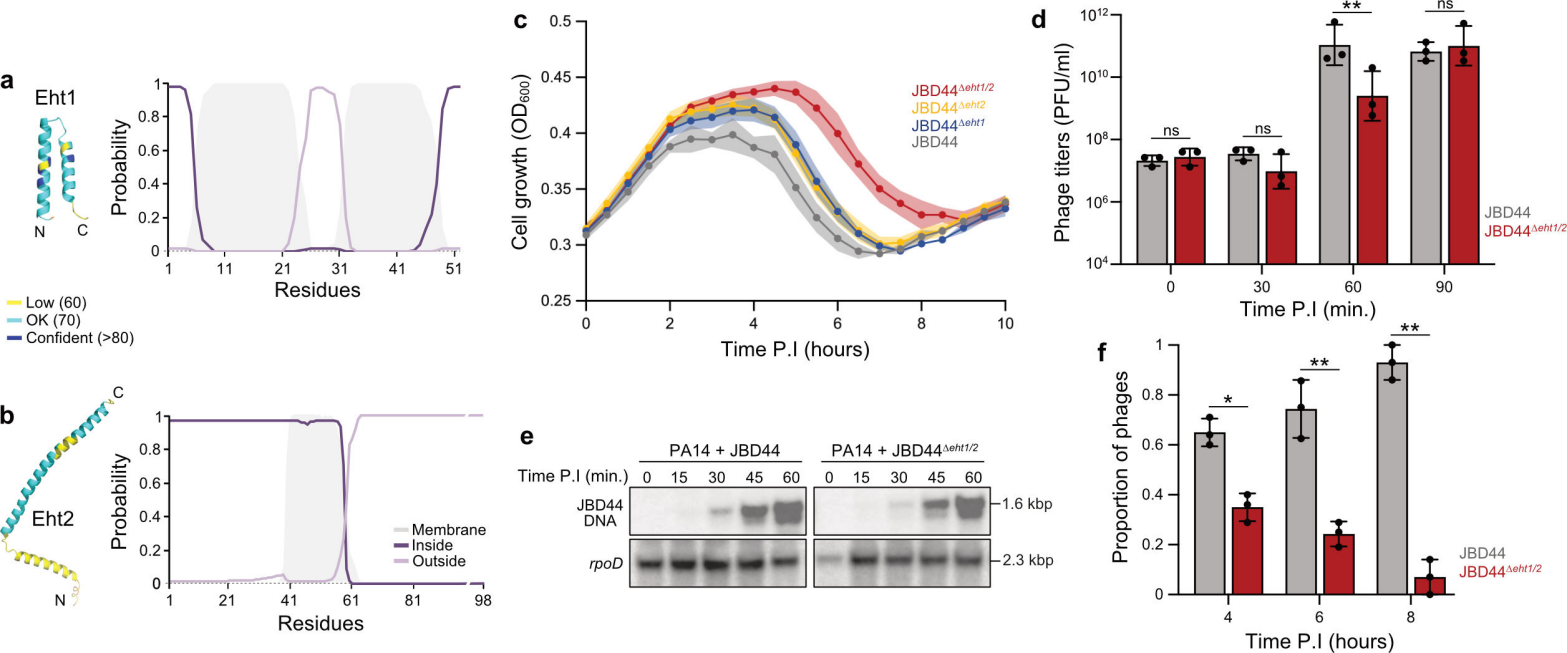
Characterization of Eht1 and Eht2. AlphaFold2 structural models with pLDDT scores denoted by color for (a.) Eht1 and (b.) Eht2 shown on the left and the predicted membrane localization (gray in membrane) using DeepTMHMM on the right. (c) Bacterial growth curves of PA14 challenged with the indicated phage. Data are presented as mean values, and the shaded regions represent the standard error across three replicates. (d) Titers of JBD44 or JBD44^Δ^*^eht1/2^* phages, measured as plaque-forming units (PFU), produced during infection of PA14. Samples were taken at 30-minute intervals. (e) Southern blot analysis for JBD44 and JBD44^Δ^*^eht1/2^* phage genome replication during infection of PA14. PA14 *rpoD* was used as a loading control. (f) Proportion of JBD44 or JBD44^Δ^*^eht1/2^* phages produced during infection of PA14. Error bars represent standard error of three replicates. Asterisks show statistically significant differences. *P < 0.05; **P < 0.01 (paired *t*-test).

As many of the differentially expressed PA14 genes upon JBD44 infection were associated with KEGG pathways related to membrane-localized signaling and transport processes, we were intrigued to find two JBD44 genes that were predicted to encode small, membrane-localized proteins. These genes, *8* (*eht1*) and *9 (eht2),* were expressed early in the infection cycle (Fig. 1a; Table S1). AlphaFold (30) and DeepTMHMM (31) predicted their products, Eht1 and Eht2 for “early host takeover” proteins 1 and 2, respectively, to be alpha-helical proteins that traverse the bacterial cell membrane. Eht1 was predicted to adopt a fold with two membrane-spanning helices where both N- and C-termini are located in the cytoplasm (Fig. 3a). Eht2 was predicted to have a single transmembrane helix that orients the N-terminus in the cytoplasm (Fig. 3b). These two proteins are the only predicted membrane-associated proteins identified in the early expressed region of phage JBD44.

To determine how often *eht1* and *eht2* are found in phage genomes, we performed BLASTN (32) searches. We found that *eht1* is conserved in the early gene operon in 139 *P*. *aeruginosa* phage genomes and ten phage genomes of other species of *Pseudomonas*, including *P. putida* and *P. umsongensis*. In all 139 *P*. *aeruginosa* phages, *eht1* is always found immediately upstream of *eht2*. By contrast, *eht1* is found alone in nine of the ten phages of other *Pseudomonas* species. When we used *eht2* as a query, we found 40 additional *P. aeruginosa* phage and prophage genomes that encode *eht2* alone. Further analyses revealed that *eht1* and *eht2* are not always present in phages that harbor a region like the phage λ right operon, such as phages D3 and PAJU2 (Fig. 1b). These results suggest an accessory role for Eht1 and Eht2, which is likely more important for phages that infect *P. aeruginosa* over other species. It also suggests that they may be functionally linked. In support of the structural predictions for Eht1, we were able to purify it from the inner membrane fraction of *E. coli* C43 cells (Fig. S1a). To determine if Eht1 interacts with Eht2, we used a bacterial adenylate cyclase two-hybrid assay (33). This assay revealed a potential interaction between Eht1 and Eht2 (Fig. S1b). Together, these data suggest that Eht1 and Eht2 may function together in *P. aeruginosa* during early phage infection.

To gain insight into the roles of Eht1 and Eht2 during phage infection, we created deletion mutants of *eht1*, *eht2,* and *eht1/eht2* in phage JBD44 using CRISPR-Cas engineering (34). We performed liquid growth infection assays where we challenged individual populations of PA14 cells in the early exponential growth phase (OD_600_ = 0.4) with wild-type JBD44 and the three mutant phages, JBD44^Δ^*^eht1^*, JBD44^Δ^*^eht2^*, and JBD44^Δ^*^eht1/2^*, each at a multiplicity of infection (MOI) of 1 and monitored cell growth for 10 hours. We found there was a faster decline in population density upon infection by wild-type JBD44 compared to JBD44^Δ^*^eht1^* or JBD44^Δ^*^eht2^*, suggesting that their replication pathways were delayed. This effect was magnified during infection by the JBD44^Δ^*^eht1/2^* double mutant (Fig. 3c). These results suggest thatthe activity of Eht1 and Eht2 is necessary for optimal phage replication. To confirm that the delayed lysis phenotype we observed for the mutant phage was due to the loss of Eht1 and Eht2 activity, we cloned *eht1* and *eht2* alone and together into expression plasmids and expressed the proteins in *P. aeruginosa* (Fig. S1c). The expression of Eht1 and Eht2, alone or in combination, did not impact bacterial growth rates in liquid media (Fig. S1d). We then repeated the infection assays and found that the expressions of Eht1 and Eht2 partially rescued the delay in JBD44^Δ^*^eht1^* and JBD44^Δ^*^eht2^* infection, respectively. In addition, expression of Eht2 partially rescued the delay in JBD44^Δ^*^eht1/2^* infection (Fig. S1e).

The delayed population-level cell lysis we observed could indicate either a longer phage replication cycle or a replication cycle that produces fewer phage progeny. To gain insight into which of these was occurring, we examined phage production in liquid media for a single round of infection. We incubated PA14 with wild-type JBD44 or JBD44^Δ^*^eht1/2^* at an MOI of 1 for 15 minutes to allow phages to adsorb. We then washed away any unabsorbed phages and incubated the cultures at 37°C with shaking to allow the phage infection cycle to proceed. We removed samples every 15 minutes up to 90 minutes post-infection and determined the number of bacterial cells and phages at each time point. We found that JBD44 produced >tenfold more phages than JBD44^Δ^*^eht1/2^* at 60 minutes post-infection (Fig. 3d). This difference in phage production disappeared by the 90 minute time point, with both cultures producing 10^7^ phages/mL, suggesting that the JBD44^Δ^*^eht1/2^* replication cycle is less efficient than that of the wild-type JBD44. We next assessed phage genome replication using the same assay. We extracted DNA from the samples at 15, 30, 45, and 60 minutes post-infection and assessed the amount of JBD44 genome present using a Southern blot. We found that phage genome replication proceeded at a similar rate in the wild-type and mutant phages (Fig. 3e), suggesting Eht1 and Eht2 activity somehow affects the speed or efficiency with which virions are assembled.

While JBD44^Δ^*^eht1/2^* produces a similar number of phage particles as JBD44, it takes a longer time to do so, which would likely be a disadvantage when phages are competing for the same host. To determine if the combined activity of Eht1 and Eht2 provides a competitive advantage, we performed a competition assay where we challenged a population of cells with both JBD44 and JBD44^Δ^*^eht1/2^*, each at an MOI of 1. Phage samples were taken at 4, 6, and 8 hours post-infection, and the proportion of each phage present at each time point was determined by PCR screening of individual plaques. We found that wild-type JBD44 accounted for 65%, 74%, and 93% of the plaques at 4, 6, and 8 hours post-infection, respectively (Fig. 3f). Therefore, the increased replication efficiency driven by Eht1 and Eht2 activity provides a competitive advantage to JBD44.

### Eht1 and Eht2 stimulate c-di-GMP signaling

**Fig 4:**
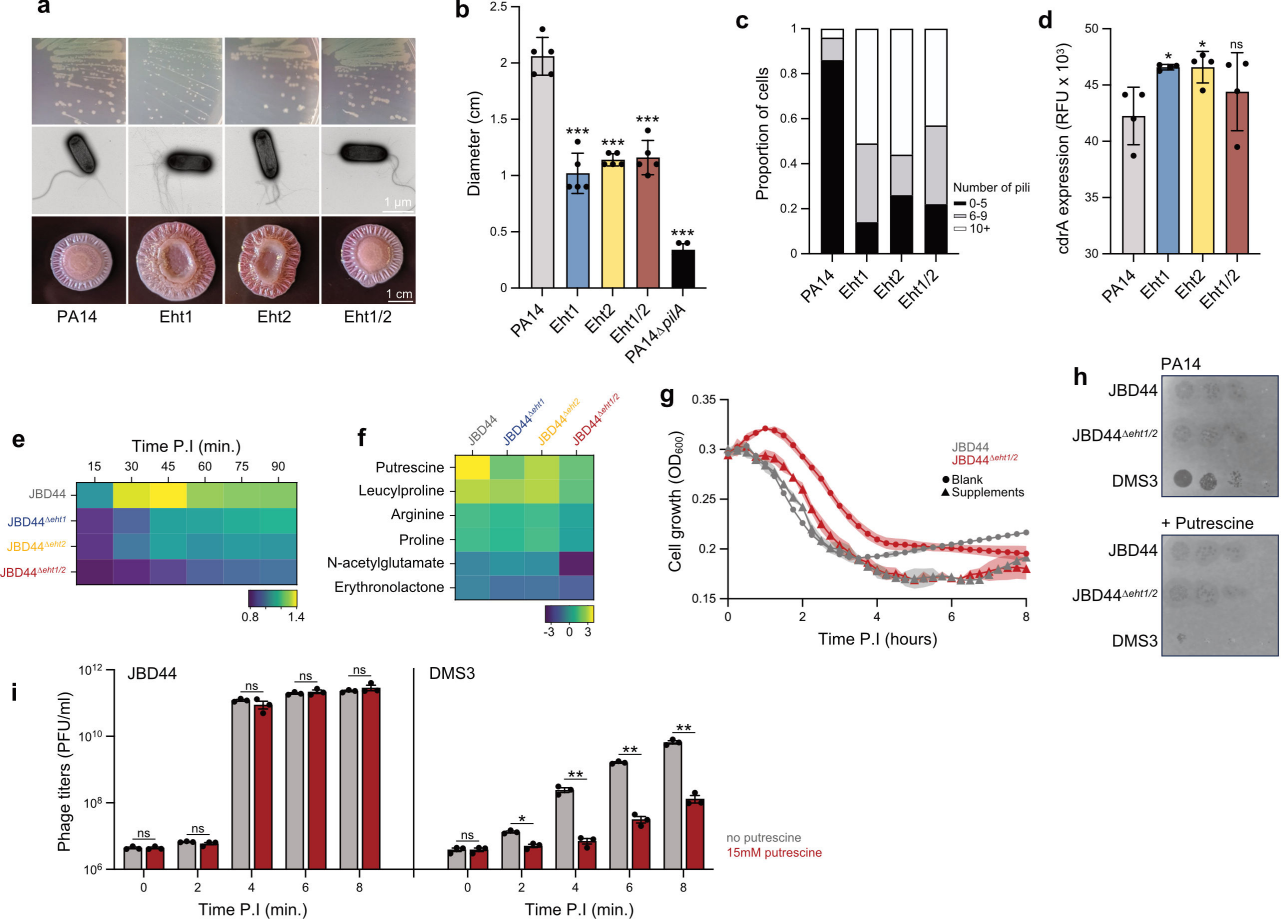
Phenotypic analyses of Eht1- and Eht2-expressing cells. (a) Phenotypes resulting from the expressions of Eht1 and Eht2. Shown are PA14 containing empty vector (PA14) or expressing Eht1 alone (Eht1), Eht2 alone (Eht2), or both (Eht1/2). Traits assessed include colony morphology (upper), surface piliation (middle), and pel exopolysaccharide production (bottom). (b) Quantification of twitching motility assays for the constructs shown in panel a. Error bars represent standard deviation of five replicates. (c) Quantification of the proportion of 100 cells expressing Eht1, Eht2, or Eht1/2 that display 0–5 (black), 6–9 (gray), or ≥10 (white) surface pili. (d) Relative fluorescence intensity from a GFP-based *cdrA* transcriptional reporter. Error bars represent standard deviation of four replicates. Asterisks show statistically significant differences. *P < 0.05; ***P < 0.001 (paired *t*-test). (e) Heat map showing the fold-change in the relative fluorescence intensity from a GFP-based *cdrA* transcriptional reporter during infection of PA14 by the indicated phage compared to uninfected cells. The number of minutes post-infection (P.I.) for each point is shown. (f) Heat map showing the fold change in levels of six metabolites following phage infection. Each square shows the relative change in the particular metabolite at 6 hours post-infection by the phage indicated on top. (g) Growth curves of PA14 challenged with JBD44 or JBD44^Δ^*^eht1/2^* in the absence (circles) or presence of supplements (arginine, proline, glutamic acid, and putrescine each at 5 mM; triangles). Data are presented as mean values, and the shaded regions represent the standard error across three replicates. (h) Tenfold serial dilutions of indicated phages on lawns of PA14 with or without putrescine (50 mM). (i) Titers of JBD44 or DMS3 phages, measured as plaque-forming units (PFU), produced during infection of PA14 with or without 15 mM of putrescine. Samples were taken at 2-hour intervals. Asterisks show statistically significant differences. *P < 0.05; ***P < 0.001 (paired *t*-test).

While Eht1 and Eht2 expression did not impact bacterial growth rates in liquid culture, we found that cells expressing Eht1 on solid media formed small colonies. This small-colony phenotype was not observed in cells expressing Eht2 alone or when Eht1 was expressed in combination with Eht2 (Fig. 4a, upper). We previously showed that the expression of Eht1 alone decreased twitching motility (35), a form of locomotion that allows bacteria to move along a solid surface. Twitching is mediated by the type IV pilus, a cellular appendage that dynamically extends and retracts. We found that the expression of Eht1 or Eht2 alone reduced twitching motility, although not to the levels observed in a PA14∆*pilA* mutant, which is unable to assemble pili (Fig. 4b). These results suggested that Eht1 and Eht2 expression alters behaviors associated with surfacing sensing and attachment.

The type IV pilus is a common cell surface receptor for *P. aeruginosa* phages, and phages have been shown to interfere with its assembly to prevent subsequent phage infection (36, 37). To gain insight into how Eht1 and Eht2 activity decreased twitching motility, we grew cells expressing Eht1, Eht2, or Eht1/2 on solid agar to promote pilus production and used negative staining electron microscopy to visualize the pili on the surface of cells (Fig. 4a, middle). We used a PA14∆*pilT* mutant strain that can assemble but not retract pili (38) to trap any assembled pili on the surface. We found that 86% of wild-type PA14 cells had <5 pili, and only 4% of cells displayed >10 pili (Fig. 4c). By contrast, 51% of cells expressing Eht1 and 56% of cells expressing Eht2 displayed >10 pili. These data suggest that Eht1 and Eht2 activity stimulates pilus assembly. Hyper-piliation was previously shown to strengthen surface adhesion and reduce twitching motility in *P. aeruginosa* (39), explaining the decreased twitching motility that we observed. Increased pilus-mediated surface adhesion in *P. aeruginosa* is associated with increased production of exopolysaccharides. To determine if the expression of Eht1 and Eht2 affects exopolysaccharide production, we grew cells expressing them on agar containing Congo Red dye, which allows assessment of Pel exopolysaccharide production (40). Eht1 and Eht2 expression resulted in rugose colonies that absorbed more Congo red dye than PA14 alone or PA14 expressing Eht1 and Eht2 together (Fig. 4a, lower). These data suggest that Eht1 and Eht2 activity stimulates the production and secretion of Pel.

A key pathway that drives coordinated cellular changes associated with a surface-associated lifestyle, including reduced motility and increased exopolysaccharide production, involves the secondary messenger c-di-GMP (41, 42). As Eht1 and Eht2 expression resulted in phenotypes consistent with a surface-adhered lifestyle, we next examined c-di-GMP levels in these cells. A commonly used marker for c-di-GMP is the expression of *cdrA*, an extracellular adhesin that is highly expressed during elevated c-di-GMP levels (43). We expressed Eht1, Eht2, or both in a PA14 *cdrA* reporter strain and monitored fluorescence intensity during cell growth. We found *cdrA* promoter activity increased by ~10% upon the expression of Eht1 or Eht2 but was not significantly changed upon co-expression of these genes (Fig. 4d). In support of these results, our RNAseq data revealed a ~2-fold upregulation of *cdrA* within 5 minutes of JBD44 infection (Table S2). To determine if changes to *cdrA* expression during infection were due to Eht1 and Eht2 activity, we infected the *cdrA* reporter strain with wild-type and mutant JBD44 phages at an MOI of 1. We found *cdrA* was upregulated ~1.4-fold by 30 minutes post-infection and remained elevated by ~1.3-fold up to 90 minutes post-infection in cells infected by JBD44 compared to uninfected cells. By contrast, *cdrA* levels remain relatively unchanged in cells infected by JBD44^Δ^*^eht1^*, JBD44^Δ^*^eht2^*, or JBD44^Δ^*^eht1/2^* at all time points tested, compared to uninfected cells (Fig. 4e). This suggests that while overexpression of Eht1 or Eht2 individually can lead to an upregulation of *cdrA*, both are important for promoting the expression of this c-di-GMP-regulated gene during infection. Taken together, Eht1 and Eht2 may have some redundancy in their activity as they can promote similar phenotypes. However, we predict that the interaction between Eht1 and Eht2 may help regulate or refine their interactions with host targets during infection.

### Eht1 and Eht2 impact arginine and polyamine metabolism during infection

Our transcriptomic data and KEGG pathway analyses revealed major changes to metabolic gene expression during JBD44 infection, and c-di-GMP levels have also been linked with metabolic changes in the cell (13). Thus, we investigated the metabolite profiles of cells during infection by JBD44 and JBD44^Δ^*^eht1/2^* using mass spectrometry. We identified 144 different compounds in these samples, which included 78 compounds for which we could ascribe a function. In cells infected by wild-type JBD44, 16 metabolites were elevated, and seven metabolites were decreased >2-fold, as compared to uninfected cells (Table S4). This group of altered metabolites consisted of predominantly compounds associated with amino acid metabolism, which is in keeping with previous studies of changes in metabolites in *P. aeruginosa* upon phage infection (3, 8). The amino acid-derived polyamine putrescine was the most elevated metabolite following JBD44 infection, increasing 16-fold as compared to uninfected cells. Arginine, proline, and leucylproline levels were all increased >2-fold following JBD44 infection. These four metabolites were also elevated in cells infected by the mutant phages, but to a lesser degree. All four were elevated in JBD44-infected cells by >2-fold compared to cells infected by JBD44^Δ^*^eht1/2^* (Fig. 4f; Table S4), suggesting that Eht1 and Eht2 play a role in their regulation. The levels of two metabolites, N-acetyl-glutamic acid, an intermediate in the arginine production pathway, and erythronolactone, used in polyamine synthesis (44), decreased ~2-fold in cells infected by JBD44 compared to uninfected cells. In addition, they were decreased by a striking 16-fold and fourfold, respectively, in cells infected by JBD44^Δ^*^eht1/2^* compared to uninfected cells (Fig. 4f; Table S4). This suggests these metabolites are downregulated in response to phage infection, and that Eht1 and Eht2 can at least partially counteract this effect. We hypothesized that the differences in metabolite changes associated with JBD44 compared to JBD44^Δ^*^eht1/2^* infection may play a role in the difference we observed in phage replication efficiency. To test this, we repeated our infection assays in minimal growth media supplemented with 5 mM of each of arginine, proline, glutamate, and putrescine. The addition of all four metabolites in combination abolished the significant difference in the timing of population density decline between JBD44 and JBD44^Δ^*^eht1/2^* (Fig. 4g).

Putrescine, the metabolite with most elevated levels upon JBD44 infection, is one of three main polyamines in bacteria, along with spermidine and spermine (45). These small cationic compounds bind to nucleic acids and have been shown to promote phage replication and genome packaging (9, 46). Intriguingly, putrescine was also recently reported to play a role in anti-phage response in *P. aeruginosa*; it was shown to be released from lysed cells and internalized by adjacent cells, where it accumulated and interfered with phage replication. This was shown to provide resistance against diverse double-stranded DNA phages as well as a single-stranded DNA phage (47). To determine if JBD44 replication was affected by putrescine, we assessed the ability of wild-type JBD44 and JBD44^Δ^*^eht1/2^* to replicate in PA14 grown on agar containing high levels of putrescine (50 mM). We found that in contrast to phage DMS3, which was previously shown to be inhibited by putrescine (47), JBD44 and JBD44^Δ^*^eht1/2^* can readily replicate in its presence (Fig. 4h). As wild-type JDB44 elicits higher production of putrescine than JBD44^Δ^*^eht1/2^*, this suggests that JBD44 stimulates putrescine production as part of a host takeover strategy and that this activity may contribute to more efficient phage replication. To determine if the ability of JBD44 to replicate efficiently in the presence of putrescine provides an advantage when phages are competing for the same host, we next examined the replication of JBD44 and DMS3 upon co-infection. We discovered that a culture co-infected with equal numbers of JBD44 and DMS3 produced approximately 30-fold more JBD44 phages following 8 hours of growth as compared to DMS3 (Fig. 4i). By contrast, in the presence of 15 mM putrescine, phage JBD44 outnumbered phage DMS3 by more than 2,000-fold (Fig. 4i). These results confirm that putrescine provides JBD44 with a competitive growth advantage against phage DMS3.

## DISCUSSION

Phages must co-opt bacterial cell metabolic processes in order to replicate. To aid in host takeover, phages express proteins immediately upon infection, which help redirect the host metabolism to benefit the phage. In this work, we identified two phage proteins, Eht1 and Eht2, that act to increase the fitness of phage JBD44 by enhancing replication efficiency. While the total number of phages produced in a single infection cycle for JBD44 and JBD44^Δ^*^eht1/2^* was equal, the wild-type phage completed the infection cycle in less time, thereby providing a competitive growth advantage. Infection by the wild-type phage resulted in an increased level of putrescine, a compound associated specifically to the arginine and proline metabolic pathway. Supplementation of the media with these key amino acids and putrescine restored the infection efficiency of JBD44^Δ^*^eht1/2^*. As putrescine has been shown to enhance the efficiency of translation and genome packaging (9, 46), this presents a likely explanation for JBD44 harnessing its activity.

While the precise mechanisms of activity for Eht1 and Eht2 were not resolved, several lines of evidence allow us to propose a model where Eht1 and Eht2 localize to the inner membrane and target two-component signaling to drive changes to the expression of metabolic genes associated with arginine and proline metabolism. In support of this, phage JBD44 infection was accompanied by many changes in host gene expression. These changes were predominantly observed in pathways associated with stress response, nitrogen metabolism, and amino acid metabolism, consistent with previous studies that examined host response to phage infection in *P. aeruginosa* (3, 27). Arginine can be broken down through different pathways. We found specific upregulation of genes involved in the catabolic pathways that result in increased levels of putrescine and downregulation of genes involved in other catabolic pathways, such as denitrification and arginine fermentation. This is consistent with our metabolic analyses that revealed elevated levels of putrescine during infection. Two-component systems have been shown to be targeted by phages (7, 48, 49), but this remains an understudied aspect of host takeover. We propose that Eht1 and Eht2 work together to fine-tune their activities as they are frequently encoded together in *P. aeruginosa* phages, their co-expression results in more subtle impacts to bacterial phenotypes compared to individual expression, and deletion of both genes results in the greatest impairment to infection efficiency.

Our work provides new insight into phage-mediated reprogramming of bacterial metabolism during host takeover. Previous studies investigating metabolic alterations of phage-infected cells found that certain pathways, including those involved in carbohydrate, nucleotide, and amino acid metabolism, are commonly affected by phage infection (8, 50). However, as different phages can impact these pathways in different ways, these studies suggested that there is no universal response to infection. Our results provide additional support for this observation, with similar but not identical pathways affected upon JBD44 infection. These results also provide important insight into how phages can employ host takeover strategies to outcompete other phages. For example, amino acid metabolism has been shown to be repressed during infection by diverse phages; Eht1 and Eht2 counteract this effect by upregulating a key amino acid pathway that prevents depletion of these virion building blocks. In addition, the replication of many other *P. aeruginosa* phages has been shown to be inhibited in the presence of putrescine (47). JBD44 is not only able to bypass putrescine-mediated inhibition, but it seems also to actively promote its synthesis for a benefit. These two elements of Eht1 and Eht2 activity could provide important growth advantages over other phages.

Many early-expressed phage accessory genes remain uncharacterized. As phages all have unique complements of these accessory genes, their infection cycles will result in unique metabolic profiles that may be difficult to predict. While some previously investigated auxiliary metabolic genes were selected as they had putative metabolic enzymatic activities, Eht1 and Eht2 had no similarity to proteins of known function and thus could not have been predicted to participate in metabolic changes. As phages are being actively investigated for therapeutic purposes, understanding how they affect host metabolism and the phage life cycle itself can provide insight into new antibacterial targets and will be key to designing effective therapies.

## MATERIALS AND METHODS

### Bacterial and phage strains and cloning procedures

Strains, plasmids, and primers are listed in Table S5. *P. aeruginosa* strain PA14 was used for all assays, unless otherwise noted. Cells were grown in lysogeny broth (LB) or M9 minimal media (20 mM NH_4_Cl, 12 mM Na_2_HPO_4_, 22 mM KH_2_PO_4_, 8.6 mM NaCl, 1 mM MgSO_4_, and 0.5% casamino acids) overnight with shaking (250 rpm) at 30°C or 37°C, as noted. JBD44 phage stocks were generated by growing a PA14-JBD44 lysogen in LB overnight at 37°C to allow spontaneous phage induction to occur. The bacterial cells and debris were collected by centrifugation, the supernatant containing the phages was dialysed into SM buffer (100 mM NaCl, 50 mM Tris-HCl [pH 7.5], and 0.01% [wt/vol] gelatin), and the lysates were stored at 4°C. All cloning procedures were done by standard restriction enzyme cloning procedures using Phusion (Invitrogen) and chemically competent *E. coli* DH5α cells. Plasmid sequences were verified by Sanger sequencing (Eurofins).

### Generating phage mutants

Gene deletions of *eht1*, *eht2,* and *eht1/2* were generated using a CRISPR-Cas3 system (34). Target sequences in *eht1* and *eht2* were used to construct CRISPR guide RNAs using the primers listed in Table S5. Repair templates lacking *eht1*, *eht2,* or *eht1/2* were synthesized (Twist Biosciences), amplified using primers listed in Table S5, and cloned into pHERD30T. PA14 strains harboring each of these plasmids were infected by wild-type JBD44 and grown in LB overnight at 30°C to allow phage replication and recombination to occur. Phages were collected from the supernatant and plated on PA14 expressing the CRISPR type I-C plasmid with guides that target *eht1* or *eht2*. Mutant phages that escaped this targeting were selected and resuspRNA sMETended in SM buffer for storage. The deletions were confirmed by Sanger sequencing (Eurofins).

### Liquid phage infection assays and phage plating assays

For liquid infection assays, PA14 was grown in LB or M9 media overnight at 37°C and then diluted 1:100 into 100 μL of fresh media with 10 mM MgSO_4_. M9 media was supplemented with 5 mM arginine, proline, glutamic acid, and putrescine, as noted. When the infection assay was performed in PA14 expressing Eht1, Eht2, or Eht1/2 from pHERD30T, cultures were grown in LB supplemented with 50 μg/mL gentamicin and 0.1% arabinose. One hundred microliters of media was aliquoted into wells of a clear 96-well plate and grown with shaking at 30°C in an Infinity microplate plate reader (Tecan) to an OD_600_ of 0.4. The indicated phages were added at an MOI of 1 and grown up to 24 hours. To perform phage plating assays, PA14 lysogens were grown in LB overnight at 37°C. One hundred fifty microliters of the culture was mixed with LB top agar (0.7% agar, 10 mM MgSO_4_) and overlayed onto LB agar plates (1.5% agar, 10 mM MgSO_4_); 2 μL of tenfold serial dilutions of phage lysates was plated on the lawn. Plates were incubated at 30°C overnight. All assays were performed in triplicate.

### JBD44 and DMS3 phage competition assays

Overnight cultures of PA14 were diluted 1:100 into fresh LB media, with and without 15 mM of putrescine, and grown to an OD_600_ of 0.4 at 37°C. The cultures were then co-infected with phages JBD44 and DMS3 at an MOI of 0.01. Phages samples were collected at 0, 2, 4, 6, and 8 hours post-infection. To quantify the phage titers, JBD44 was selected for on a lawn of PA14 Δ*pilA,* and DMS3 was selected for on a lawn of PA14 Δ*wbpL*. Graphs and statistical analyses were performed in GraphPad Prism 9.0.2.

### RNA extraction, sequencing analysis, and data visualization

PA14 was grown in LB overnight at 37°C, then diluted 1:100 into 2 mL of fresh LB supplemented with 10 mM MgSO_4_, and grown at 30°C until an OD_600_ of 0.4 was reached. Phages were added to the bacterial cultures at an MOI of 10 to ensure synchronous infection, and samples were collected at various time points post-infection. Cells were collected by centrifugation (21,000 × *g*), and organic RNA extraction was performed as previously described (51). Small RNAseq library preparation and NovaSeq 6000 SP (Illumina) RNA sequencing were performed at the Donnelly Sequencing Center (Toronto, ON) to generate trimmed, demultiplexed, single-end reads (1 × 100 bp read length) with an average read depth of 20M. The assay was performed in two replicates. Quality control using FastQC was completed, which confirmed all reads had minimum and average Phred33 scores of 20 and 36, respectively. Data were collectively analyzed using the UseGalaxy interface and following the Galaxy protocol for reference-based RNAseq data analysis (long) (52). The reads were mapped with BWA-MEM (53) against the phage JBD44 genome, counted using featurecounts (54), and differential expression was calculated using DeSeq2 (55). Differential expression of genes is shown for JBD44 (Table S1) and PA14 (Table S2). The ClueGO plugin of Cytoscape software was used to group differentially expressed PA14 genes based on the KEGG genes database (K-score threshold = 0.4, *P* < 0.05 using the Fischer Exact Test, minimum three genes in a cluster and a sharing group percentage of 30) (28).

### Phage titer assays and Southern blots

PA14 was grown in LB overnight at 37°C, diluted 1:100 into 2 mL of fresh media supplemented with 10 mM MgSO_4_, and then grown at 30°C until an OD_600_ of 0.4 was reached. Phages were added to the bacterial cultures at an MOI of 1, and cells were collected by centrifugation (21,000 × *g*) at various time points post-infection. To determine phage titers, the supernatant was collected, and a phage plating assay was performed. For the competition assay, specific primers were used to screen individual plaques. For the Southern blot, bacterial pellets were flash-frozen and stored at −80°C. Total DNA of both bacteria and phages was isolated using the Qiagen DNeasy Kit (Qiagen; 69504). The purified DNA samples were digested with *Xho*I and *Not*I and separated on a 1% agarose gel at 150 V in TAE buffer (40 mM Tris-acetate, pH 8.3, 1 mM EDTA). DNA was transferred to a nylon membrane (Hybond-N+; Amersham) using the capillary transfer method and fixed by baking the membrane at 120°C for 30 minutes. Digoxigenin (DIG-labeled) nucleic acid probes specific to the JBD44 large terminase (gene *18*) and bacterial *rpoD* genes were generated by random prime labeling PCR fragments using primers listed in Table S1. DNA hybridizations were performed at 42°C overnight using the DIG Easy Hyb solution (Roche), and unbound probes were washed off using a high stringency buffer (0.1× SSC containing 0.1% SDS; Roche) at 68°C. DIG-labeled probes bound to DNA were detected using anti-DIG-alkaline phosphatase antibody (Roche; 1:10,000 dilution in 1 × Blocking Solution), developed using 0.25 mM CDP-*Star* solution (Roche), and imaged with the Bio-Rad ChemiDoc Imaging system. Capillary transfer, probe hybridizations, and probe detection with chemiluminescence were performed according to the Roche DIG application manual for filter hybridization using the DIG-High Prime DNA Labeling and Detection Starter Kit II (Roche; 11585614910). The assay was performed in triplicate.

### Protein expression tests and inner membrane protein purification

*eht1* and *eht2* were cloned into the petDuet-1 vector under the T7 promoter in-frame with an N-terminal 6×His-tag using primers listed in Table S1. For expression tests in PA14, 6×His-tagged Eht1 and Eht2 were subcloned into pHERD30T. PA14 cultures expressing these proteins were grown in LB with 50 μg/mL gentamicin and 0.1% arabinose to an OD_600_ of 0.8. Whole cell lysate was collected, and a Western blot was performed to visualize whole cell protein expression. For inner membrane purification, *E. coli* C43 cells expressing the Eht1 pETDuet-1 construct were grown in LB with 100 μg/mL ampicillin at 37°C to an OD_600_ of 0.8. Then, 1 mM IPTG was added to the culture to induce expression, followed by overnight incubation with shaking at 16°C. Cells were collected by centrifugation (21,000 × *g*), and the cell pellet was resuspended in lysis buffer (50 mM Tris-HCl [pH 7.5], 100 mM NaCl, 1% Triton X-100, 10 mg/mL DNase and 1 complete mini, EDTA-free Protease Inhibitor Cocktail tablet [Roche]). The cells were lysed by sonication, and the membrane fractions were isolated by ultracentrifugation (72,000 × *g*) for 1 hour. The pellet was solubilized by resuspension in solubilization buffer (20 mM Tris [pH 8], 2% Triton X-100, and 10 MgCl_2_) normalized to cell pellet weight. All fractions were resuspended in sample loading buffer and incubated at 100°C for 5 minutes, except the membrane fractions, which were incubated at 50°C for 15 minutes to avoid aggregation. Proteins were separated by SDS-PAGE and visualized with a Western blot using anti-His antibody probes.

### Bacterial adenylate cyclase two-hybrid assay

Genes *eht1* and *eht2* were cloned into pUT18C or pKT25 plasmids to be fused with the T18 and T25 domains of CyaA adenylate cyclase using primers listed in Table S1. Sequence-verified plasmids were co-transformed into *E. coli* strain BTH10 for the assay (56). Single colonies were inoculated into LB with 50 μg/mL kanamycin and 100 μg/mL ampicillin and grown overnight at 30°C. The following day, 2 μL of the culture was spotted onto MacConkey plates (1.5% agar, 50 μg/mL kanamycin, 100 μg/mL ampicillin, 0.5 mM IPTG, and 1% maltose) or LB X-gal plates (1.5% agar, 50 µg/mL kanamycin, 100 µg/mL ampicillin, 0.5 mM IPTG, and 40 µg/mL X-gal). Plates were incubated at 30°C for 24 to 48 hours. Positive interactions reconstituted adenylate cyclase and resulted in a color change to red on MacConkey agar or blue on LB agar plates containing X-gal. The assay was performed in triplicate.

### Colony morphology assays

PA14 expressing *eht1*, *eht2,* or *eht1/2* from pHERD30T was streaked onto *Pseudomonas* isolation agar plates (1.5% agar, 50 μg/mL gentamicin, and 0.1% L-arabinose) and incubated for 48 hours at 30°C. For the Congo red assay, single colonies were grown overnight at 37°C in M9 minimal media supplemented with 50 μg/mL gentamicin and 0.1% arabinose. Two microliters of the culture was plated on Congo red agar plates (1.5% agar, 10 g/L tryptone, 40 μg/mL Congo red, 20 μg/mL Coomassie brilliant blue, 50 μg/mL gentamicin, and 0.1% arabinose) and incubated at 30°C in the dark for 3 days. The assay was performed in triplicate.

### Twitching motility assays

Single colonies of PA14 expressing *eht1*, *eht2,* or *eht1/2* from pHERD30T were inoculated onto LB agar plates (1% agar, 50 μg/mL gentamicin, and 0.1% arabinose) and incubated for 24 hours at 30°C. The agar was removed, 1% crystal violet was used to stain the biomass adhered to the Petri plates, and the diameter was measured to determine the distance traveled. The assay was performed in triplicate.

### Visualization of pili by transmission electron microscopy

Single colonies of PA14∆*pilT* expressing *eht1*, *eht2,* or *eht1/2* from pHERD30T were streaked onto LB agar plates (1.5% agar, 50 μg/mL gentamicin, and 0.1% arabinose) and incubated for 24 hours at 37°C. The following day, single colonies were resuspended in 20 μL of SM buffer, and 7 μL was spotted onto a glow-discharged carbon-coated copper grid (CF400-CU, 6 nm, 400 mesh, Electron Microscopy Sciences). Samples were allowed to adsorb for 2  minutes before excess culture was gently removed onto filter paper, and grids were washed twice with filtered distilled water. The grids were negatively stained with 15 μL of 2% uranyl acetate for 15  seconds. Grids were imaged with a JEM-1011 (JEOL USA, INC.), digital CDD camera (5 megapixels XR50S, AMT, USA).

### Transcriptional reporter assays

We engineered a PA14 strain where green fluorescent protein (GFP) expression was under the control of the *cdrA* promoter (57, 58). Successful Tn7 integration was confirmed by Sanger sequencing. For the *cdrA* promoter plasmid assay, PA14 Tn7::PcdrA-GFP(ASV)C cells were transformed by pHERD20T plasmids expressing *eht1*, *eht2*, or *eht1/2*. Cells were grown overnight at 37°C in LB with 300 µg/mL carbenicillin, diluted 1:100 in fresh media containing 300 µg/mL carbenicillin and 0.1% arabinose, and then 100 µL was added to black-walled, clear-bottom 96-well plates (Costar). Cells were grown with shaking at 37°C in a BMG Labtech CLARIO star plate reader. GFP fluorescence readings were taken every 15 minutes and normalized to cell density (OD_600_). For the *cdrA* promoter infection assay, PA14 Tn7::PcdrA-GFP(ASV)C cells were grown overnight at 37°C in LB, diluted 1:100 in fresh media, and 100 μL was added to black-walled, clear-bottom 96-well plates (Costar). Cells were grown with shaking at 37°C in a BMG Labtech CLARIO star plate reader to an OD_600_ of 0.3, and phages were added at an MOI of 10. GFP fluorescence readings were taken every 15 minutes and normalized to OD_600_. The assays were performed in triplicate.

### Metabolomics using liquid chromatography mass spectrometry

PA14 was grown in LB overnight at 37°C, diluted 1:100 into 2 mL of fresh media with 10 mM MgSO_4_, and grown at 30°C to an OD_600_ of 0.4. Phages were added to the bacterial culture at an MOI of 1, and samples were collected at 6 hours post-infection and were immediately added to cold extraction buffer (0.1 M formic acid in MeOH:acetonitrile:H_2_O [40:40:20]) and incubated at −20°C for 30 minutes. After three rounds of centrifugation (21,000 × *g*), the supernatant was removed and resuspended in fresh extraction buffer, and the supernatant from each round was pooled. Cold ^15^N NH_4_OH was added to neutralize the sample, and samples were stored at −80°C. Samples were dried by vacuum centrifugation and stored at −80°C before being sent for untargeted mass spectrometry (59) at the BioZone, University of Toronto (Toronto, ON, Canada). Compounds were annotated using ChemSpider, ChemicalFormula, and mzCloud. Compounds within the error limit of 5 ppm (annotated delta mass) were selected for further analysis. The peak area ratio was normalized to the OD_600_ of the cells at the time of extraction. The assay was performed in two replicates. The KEGGScape plugin of the Cytoscape software was used to group pathways of differentially expressed PA14 compounds into KEGG pathways using default parameters (60).

## SUPPLEMENTAL MATERIAL

Figure S1 mbio.02466-24-s0001.tiff Western blot, bacterial two-hybrid assay, and bacterial growth curves.

Supplemental Legend mbio.02466-24-s0002.docx Legend for Fig. S1.

Supplemental Tables mbio.02466-24-s0003.xlsx Tables S1-S5.

ASM does not own the copyrights to Supplemental Material that may be linked to, or accessed through, an article. The authors have granted ASM a non-exclusive, world-wide license to publish the Supplemental Material files. Please contact the corresponding author directly for reuse.
