# Supplementary figures and images for: Phage reprogramming of Pseudomonas aeruginosa amino acid metabolism drives efficient phage replication
Source: mBio. 2025 Feb 7;16(3):e02466-24. doi: 10.1128/mbio.02466-24 (PMC11898732; doi:10.1128/mbio.02466-24)

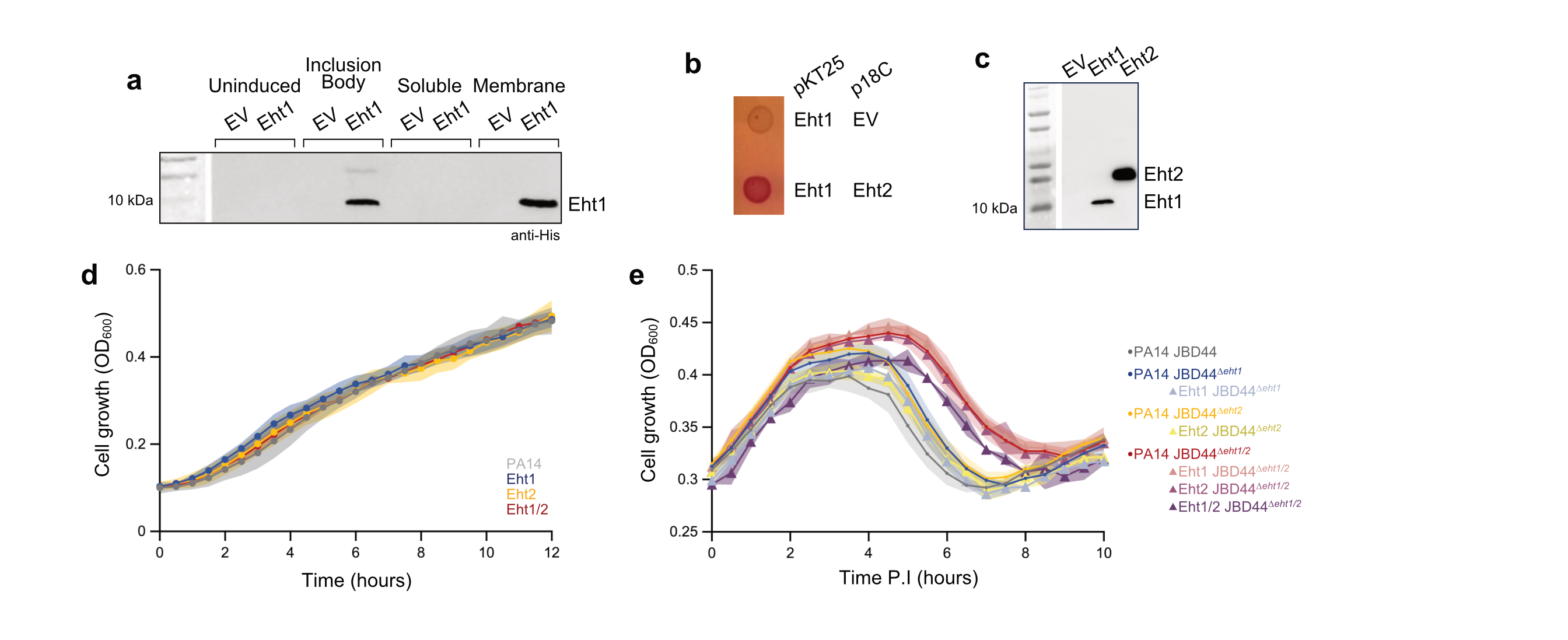

Supplement: Figure S1 — Western blot, bacterial two-hybrid assay, and bacterial growth curves. [file mbio.02466-24-s0001.tiff]
