# Supplementary material for: Phage reprogramming of Pseudomonas aeruginosa amino acid metabolism drives efficient phage replication
Source: mBio. 2025 Feb 7;16(3):e02466-24. doi: 10.1128/mbio.02466-24 (PMC11898732; doi:10.1128/mbio.02466-24)
Supplement: Supplemental Legend — Legend for Fig. S1. [file mbio.02466-24-s0002.docx]

**Supplementary Figure 1. a**. Western blot assessing membrane association of 6xHis-tagged Eht1 expressed in *E. coli*. **b**. Bacterial two hybrid assay showing interaction between Eht1 and Eht2 as indicated by the red colour change on MacConkey agar. The assay was performed in triplicate. **c**. Western blot assessing expression of 6xHis-tagged Eht1 and Eht2 in PA14. **d**. Bacterial growth curves monitoring the optical density of PA14 expressing empty vector (PA14), or expressing Eht1 alone (Eht1), Eht2 alone (Eht2), or both (Eht1/2) up to 12 hours. **e**. Bacterial growth curves of PA14 expressing empty vector (PA14), or expressing Eht1 alone (Eht1), Eht2 alone (Eht2), or both (Eht1/2) challenged with the indicated phage. Data are presented as mean values, and the shaded regions represent the standard error across three replicates.
